# An Artificial Neural Network Assisted Dynamic Light Scattering Procedure for Assessing Living Cells Size in Suspension

**DOI:** 10.3390/s20123425

**Published:** 2020-06-17

**Authors:** Dan Chicea

**Affiliations:** Research Center for Complex Physical Systems, Faculty of Sciences, “Lucian Blaga” University of Sibiu, Dr. Ion Ratiu str. no. 5–7, 550012 Sibiu, Romania; dan.chicea@ulbsibiu.ro

**Keywords:** artificial neural network, dynamic light scattering, simulated time-series, fermentation, *Saccharomyces cerevisiae*

## Abstract

Dynamic light scattering (DLS) is an essential technique used for assessing the size of the particles in suspension, covering the range from nanometers to microns. Although it has been very well established for quite some time, improvement can still be brought in simplifying the experimental setup and in employing an easier to use data processing procedure for the acquired time-series. A DLS time series processing procedure based on an artificial neural network is presented with details regarding the design, training procedure and error analysis, working over an extended particle size range. The procedure proved to be much faster regarding time-series processing and easier to use than fitting a function to the experimental data using a minimization algorithm. Results of monitoring the long-time variation of the size of the *Saccharomyces cerevisiae* during fermentation are presented, including the 10 h between dissolving from the solid form and the start of multiplication, as an application of the proposed procedure. The results indicate that the procedure can be used to identify the presence of bigger particles and to assess their size, in aqueous suspensions used in the food industry.

## 1. Introduction

When an incident light beam has a fluid containing suspended particles as target, each particle scatters light and therefore becomes a secondary light source as elastic or, more precisely, quasi-elastic scattering occurs. The particles become scattering centers (SC). If the light source is coherent the scattered waves are coherent, as well, therefore, they will interfere in the far-field. The particles in suspension undergo a complex motion, which confers a dynamic character to the far interference field, giving it the aspect of “boiling speckles”. Several articles investigated the variation of physical parameters, such as the average intensity, speckle size and speckle contrast, with the size and the concentration of the SCs, references [1,2,3] being an example. Using the variation of the above mentioned parameters for particle sizing in dynamic processes where both the SC diameter and number of SCs can change in time is not a good choice [3]. The physical method that uses the dependence between the speckle dynamics and the particles diffusion caused by the Brownian motion is called dynamic light scattering (DLS), traditionally known as photon correlation spectroscopy (PCS) The physical explanation of the method can be found in many works, such as [4,5,6,7,8], to name just a few.

DLS is widely used to analyze the size and size distributions of nanoparticles, colloids and proteins in the suspension of various solvents [9]. The DLS technique has been shown to be appealing for biomedical applications [10], such as studying homogeneity of proteins, ribonucleic acid (RNA), and their complexes as it has certain advantages over other experimental methods. Some other methods that are used in the type of investigations mentioned above include analytical ultra-centrifugation, which requires a bigger amount of sample; small angle X-ray scattering (SAXS), which requires a longer acquisition time, in the order of hours in lack of a synchrotron; and static light scattering, which again requires time averaging to compensate for the dynamic light scattering effect. The DLS technique can be used for investigating suspensions in a wide range of sample buffers, over a relatively big range of temperatures and concentrations, as well. Moreover, DLS requires very small sample amounts, even of the order of 10 μL [10] and has the advantage of providing absolute rather than relative results; therefore, it does not require calibration.

The DLS technique has been established for quite some time [4]. In the early days of DLS, photomultiplier tubes were used as detectors, as they have a fast response with good amplification [7]. Later on, photomultiplier tubes were replaced with avalanche photodiodes [7]. P-I-N diodes represent a step forward [9]. Autocorrelators contained the hardware to compute the autocorrelation function of the DLS time-series and is another typical part of the DLS setup. A laser diode can be used as a coherent light source and a data acquisition system can be used to record the DLS time-series on a computer, which can be used later to process it. Some examples of very simple experimental setups can be found in many papers, [11,12] being just some of them.

The DLS technique can still be improved by making it faster regarding data processing. The artificial neural networks (ANNs hereafter) concept is quite well established and described comprehensively in many works like [13,14], to mention just a few of them. The ANN is computer code that imitates the structure of a brain in its functioning. The code describes artificial neurons and the connections between them, by means of transfer functions and biases. ANNs appear as an appealing alternative to numerical minimization of functions in DLS time-series processing, because they can be much faster, as signal processing in an ANN is done by matrix multiplication and addition, rather than many function and numerical gradient evaluations.

ANNs have been used for data processing in optics and several papers reporting it are presented further on. Reference [15] describes an ANN for detecting amino acids and several solid organic compounds. The work in [16] explains how a trained ANN was successfully used in assessing the size and the refractive index. The paper cited as [17] describes how an ANN was used in measuring the radius of spherical particles. Reference [18] explains how the authors used an ANN trained for pattern recognition to detect the presence of hazardous fibers in the air. Reference [19] reports on using a circular ANN for measuring the particle size and refractive index.

ANNs were successfully used in processing DLS time-series as well. The work reported in [20] used an averaged scattered light intensity frequency spectrum as input for a three artificial neuron layer ANN that produced the average diameter of the suspended particles, in a narrow size range of up to 350 nm. Reference [21] reports a direct continuation of the work in [20] and describes using the autocorrelation of the recorded DLS time-series as input in a three artificial neuron layer ANN, which has the average diameter of the suspended particles as output. The range of particle size was extended up to 1200 nm. Both ANNs reported in [20,21] proved to be several thousand times faster than fitting either the Lorentzian line to the frequency spectrum [20] or the autocorrelation [21] with very small relative errors as compared to the reference method of fitting functions. 

Another example of using ANN in processing Rayleigh light scattering data for protein detection is reported in [22] with a 3-5-1 neuron structure. The work published in [23] reports on real-time determination of the total concentration of various oils and mixtures in water using ANNs. A recent work [24] uses the same input as [20], which is the averaged frequency spectrum of the DLS time signal and process it using a 5 layer ANN to produce the particle size distribution, on a small particle size range of 1–500 nm, with big errors for small particles though.

The work reported in this paper is a continuation of the previous work in [20,21] done to extend the size range of sizing particles up to 6000 nm with very good precision.

An application of using the ANN assisted DLS in detecting the presence of bigger particles in an aqueous suspension is presented, as well as a proof of concept. Such bigger particles, as compared with the solvent molecules, water in this case, or the molecules of glucose or fructose, frequently present in fermentation processes, can be bacteria with the size in the range starting from 0.2–0.4 μm [25], with a common size of several microns and reaching sizes as big as 500 and 750 μm [26]. Yeast cells can also be similarly large particles is suspension. While the presence of big particles, like several micron sized bacteria and yeast cells, can be confirmed by optical microscope examination of the suspension sampled from the fermentation environment and stretched as thin film on a glass slab; the presence of particles smaller than one micron cannot be made evident with a conventional microscope, as it is smaller than the wavelength of visible light. Under these circumstances, using a technique that is robust and that does not require calibration would be preferable. DLS appears to be suited for such measurements and ANN assisted DLS appears to be a good choice, as data processing is less computation intensive and easier to use, as highlighted in [20,21].

Yeasts are eukaryotic microorganisms and they are classified as fungi. Reference [27] describes approximately 1500 species of yeasts Yeasts have been widely used for alcohol related brewing and fermentation according to archaeological evidence [28,29,30]. The main fermentation is the *Saccharomyces* complex [31]. It contains one of the most important species for the food industry, *S. cerevisiae,* which is the agent used in wine, bread, beer, and sake fermentation. New technologies and new outcomes in bio-engineering and genetics placed *S. cerevisiae* as a model for eukaryotic biology [32]. *S. cerevisiae* became a valuable alternative for diverse chemical production [33], for functional foods, pharmaceuticals, and biofuels [34,35]. As *Saccharomyces cerevisiae* usage in fermentation is so widespread, the proposed ANN assisted DLS time series processing procedure was tested on fermentation produced by this type of yeast to detect the presence of big cells in suspension and to assess the variation of the cell size as fermentation carries on.

The next sections of this paper will present the sample preparation procedure, the procedure for generating the set for training the ANN, the results of the ANN on simulated data, and the results of the yeast cells size variation in time during a fermentation process, indicating that the ANN assisted DLS can be used as an almost real-time procedure for detecting the presence of big particles in suspension, as bacteria or yeast, and for monitoring fermentation by assessing the average size of the suspended yeast cell during the fermentation process.

## 2. Materials and Methods

### 2.1. Diluted Yeast Suspension

First an amount of 1 g of *Saccharomyces cerevisiae* in fresh, solid form was added in 10 mL of water and allowed to dissolve, which is to have the individual cells separated from each other, forming a nontransparent suspension. 

While dissolving carried on, a concentrated table sugar was prepared. Sucrose is the common sugar and it is a disaccharide, a molecule composed of two monosaccharides: glucose and fructose. Sucrose has the molecular formula C_12_H_22_O_11_ [36]. The sucrose syrup concentration was 15% weight in water. A volume of 0.2 cm^3^ of yeast suspension was added in 3.5 cm^3^ of syrup in a cuvette that was the target of a laser beam. The cell concentration was chosen in this range after several trials, to ensure the transparency of the suspension.

Time series were recorded at equal time intervals during an experiment. The temperature was maintained constant at 22 °C during the experiment. The time series were processed using the ANN assisted dynamic light scattering procedure and provided the average diameter of the particles that scattered light, as will be presented further on.

### 2.2. The Reference DLS Procedure

The DLS technique has been developed for many decades [4,5,6,7,8], and is quite well established, therefore the theory behind it is not repeated here, but the experimental setup and the main steps in assessing the average particle size using DLS are presented briefly in the following paragraphs.

**Figure 1:**
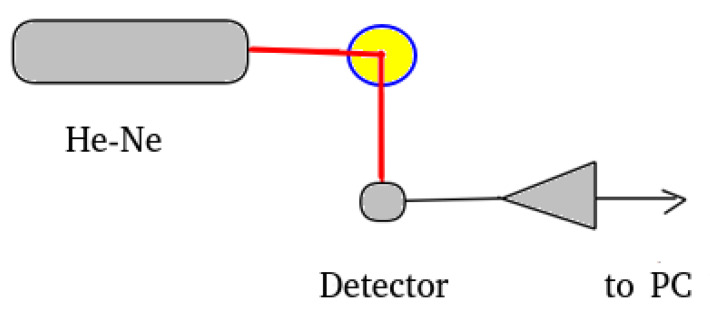
Dynamic light scattering (DLS) experimental setup.

The experimental setup is presented in Figure 1 and consists of a laser source, (a He-Ne laser, for the work reported here) with a power of 10 mW working in a continuous regime and with a wavelength of 633 nm. A 5 mL circular glass tube was used as the sample container. A detector, a preamplifier with a linear response in the audio frequency range, and a simple, single processor computer were used for recording the time-series. The scattering angle was chosen to be 90°, which is typical for DLS experiments [4,5,7,8,16].

The speckle size was measured using a Philips CCD with the optical system removed, knowing the size of the pixel on the conversion matrix. The average speckle size was assessed following the procedure presented in [3]. The cuvette-detector distance was adjusted in such a manner that the diameter of the detector was 11 cm and equaled the average speckle size.

As the incident light wave is coherent, the scattered waves are coherent as well, therefore it will interfere. What is actually measured using a detector is the intensity, not the electric field of the light wave in the detector location. The detector converts the interference field intensity to an electric signal with the voltage proportional to the intensity and the data acquisition system (DAS hereafter) converts it to an integer number in a range covered by 16 bits, and this is the DAS resolution used in this work. The data acquisition rate was 16 kHz, which was big enough for the particle range covered in this work.

The DAS produces a sequence of values recorded at equal time intervals Δt, as in Equation (1) and such a succession is called a time-series:

(1) I(0),I(Δt),I(2Δt),I(3Δt)…

In the general case, if the suspension contains m different types of particles, each having the diffusion coefficient *D*1, *D*2, …., *D*m, the intensity autocorrelation (ACR hereafter) *G*(τ) has the form [9,19]:

(2) $$G\left(\tau \right)=\sum_{i=1}^{m}A_{i}e^{-2D_{i}q^{2}\tau }+B$$

where the amplitude factors *A_i_* are proportional to the contribution of each particle group, *q* is the modulus of the scattering vector [7] detailed in Equation (3), and B is a constant.

(3) $$q=\frac{4\pi n}{\lambda }\sin\left(\frac{\theta }{2}\right)$$

In Equation (3) n is the refractive index of the solvent, *λ* is the wavelength of the incident coherent light in a vacuum, and *θ* is the scattering angle; the values of the last variables being mentioned at the beginning of this Section.

If the suspended particles can be considered a single size group, or if they have a relatively narrow distribution and can be considered as a single group, the ACR plot is very close to a simple exponentially decreasing curve. In this case the ACR in Equation (2) can be described by a simplified form, as in Equation (4):

(4) $$G\left(\tau \right)=Ae^{-2Dq^{2}\tau }+B$$

The ACR can be further normalized in two small steps. First, constant B is subtracted. The second step involves an experimental adjustment in such a manner that the detector will cover a single speckle, as described in detail in [18,19], making the spatial coherence factor included in A for this particular case, equal to 1. With this two-step normalization, for a suspension containing one group of particles with a relatively narrow particle size distribution, as stated above, the intensity ACR has a simple form depending on a single parameter, which is *D*, the diffusion coefficient:

(5) $$G\left(\tau \right)=e^{-2Dq^{2}\tau }$$

In its turn, the diffusion coefficient *D* is related to k_B_, which is Boltzmann’s constant, to η, the dynamic viscosity coefficient of the solvent, T the absolute temperature of the sample, and the hydrodynamic diameter of the particle, d, as described by the Einstein–Stokes relation [37] (6):

(6) $$D=\frac{k_{B}T}{3\pi \eta d}$$

This procedure was used as the reference for determining the average diameter of the SCs by fitting Equation (5) with *D* substituted from Equation (6) to each of the generated time-series that were used for training the ANN, as described in the next Section.

### 2.3. The ANN Assisted DLS Time-Series Processing Procedure

A brief description of the procedure, highlighting the differences and the improvement brought to the previous version, presented in [21], will be presented further on.

Training the ANN was performed with ACR of the simulated time-series as inputs and the corresponding diameters, computed using a nonlinear minimization procedure of Equation (5), as described at the end of Section 2.2, as targets. Each time-series was computed as the sum of harmonic functions, as in Equation (7):

(7) $$x\left(t\right)=\sum_{i=1}^{\mathrm{Nf}}A\left(f_{i}\right)\cdot \sin\left(2\pi f_{i}t+\varphi_{i}\right)$$

In Equation (7), *A*(*f_i_*) is the amplitude of the *i*-th component, *f_i_* is the frequency of the *i*-th component, *φ_i_* is the initial phase of the *i*-th component, *t* is the time when we compute that particular data in the DLS time-series and *N_f_* is the number of frequencies used in generating the time-series. *A*(*f_i_*), the amplitude of the *i*-th harmonic component, is computed with Equation (8), the Lorentzian line, which describes the frequency spectrum of the intensity of the scattered light, while the *φ_i_* initial phases were generated using random numbers with uniform distribution in (0, 2*π*). The frequencies of all the harmonic components *f_i_* were generated equally spaced in the interval (0, *f_s_*/2), as pointed out in [21], where *f_s_* is the sampling frequency of the DAS.

(8) $$S\left(f\right)=a_{0}\frac{a_{1}}{{\left(2\pi f\right)}^{2}+a_{1}^{2}}$$

The *a*_0_ parameter is selected to be a fixed value, of the order of tens, the same for all series, while a_1_ is calculated from Equation (9) [11,12], with *q* calculated using Equation (3) for each particle diameter *d*. *A*(*f_i_*) was selected to be the square root of *S*(*f_i_*) computed with Equation (8) [4,7,11,12].

(9) $$d=\frac{2k_{B}Tq^{2}}{3\pi \eta a_{1}}$$

where *q* is computed with Equation (3).

Rather than filtering the experimental time-series, the alternative of training the ANN with noisy time-series was chosen. As the power grid operates at 50 Hz, this component and its harmonics are present in the recorded time-series, therefore noise was added to the generated time-series, consisting of a sum of sine functions, as in Equation (9), having frequencies of type 50 × *i*, *i* being a natural number in the range 1–*i_max_*. where *i_max_* is the sampling frequency divided by 50 and then rounded, therefore it is the maximum frequency of the 50 Hz harmonic that is smaller than f_s_. The amplitudes of the 50 Hz noise harmonics decrease exponentially with the number of the harmonic, as in Equation (10), where *A_ts_* is the amplitude of the time-series, assessed as the difference between the maximum and the minimum of that particular time-series and *i* is the harmonic number. The initial phases of the harmonics *φ_i_* were generated using random numbers with uniform distribution in (0, 2*π*). The power grid noise time-series *x_h_* was computed apart from the time-series and added to it, as in [21].

(10) $$x_{h}\left(t\right)=\sum_{i=1}^{i_{max}}0.03\cdot A_{ts}\cdot \exp\left(-0.25\cdot i\right)\cdot \sin\left(2\pi f_{i}t+\varphi_{i}\right)$$

In addition to the 50 Hz noise, a random noise was added as well, and the time-series, *x_noise_*, was computed apart from the time-series using Equation (12), where *N_rnd_* is the number of frequencies f_i_ generated using random numbers with uniform distribution in the range [1–f_s_]. For the work reported here *N_rnd_* was selected to be 300.

(11) $$x_{noise}\left(t\right)=\sum_{i=1}^{N_{rnd}}0.01\cdot A_{ts}\cdot \exp\left(-0.005\cdot i\right)\cdot \sin\left(2\pi f_{i}t+\varphi_{i}\right)$$

After computing the noise time series with the power grid noise and with the random noise, these series were added to the generated time-series to produce the (realistically) noisy time-series. 

The set of time-series was generated with 32,768 data points each, with a number of 16,385 frequencies in Equation (7) and with noise added as described in the previous paragraphs of this Section. A detailed analysis that led to the selection of values as described above is presented in detail in Section 2.3 of [21], therefore not repeated here. The coefficients in Equations (10) and (11) were selected after a process of trial and error, aiming to describe the noise that is present in experimentally recorded DLS time series as realistically as possible. It is the intention both to extend the range of average diameters that can be assesses using the ANN assisted DLS procedure, and to improve the precision, the reason the ANN procedure was revisited.

The targets (diameters corresponding to each generated time series) were computed using the reference method of fitting Equation (5) with D replaced from (6) on the ACR rather than fitting Equation (8) on the frequency spectrum, as in [21]. Fitting a function to data requires numerical gradient evaluations, with the number of floating point operations increasing with the number of free parameters, therefore fitting a function with one free parameter, as described by Equation (5) requires less floating point operations than fitting a function with two free parameters, as the function in Equation (8). Moreover, computing the power spectrum using the fast Fourier transform algorithm has the disadvantage of requiring a number of 2^n^ data in the time series, otherwise it adds zeros to match the amount. The addition artificially raises the small frequency part of the spectrum, producing a fake turnover point in the very low frequency of the spectrum, therefore indicating a much bigger diameter that the real one.

Another improvement, as compared with the procedure described in [21], is that the diameters range of the simulated time series was 25–6000 nm instead of 10–1200 nm.

One time-series with noise added to it was generated for each diameter. The step for increasing the diameter was 0.5 nm.

The ANN is a feed-forward type with three layers. The input to the ANN is the autocorrelation of the time-series, more precisely the first 350 lags. Consequently, the input layer has 350 neurons, each corresponding to a value from the ACR data set. The hidden layer has 26 neurons. The output layer has one neuron, as the output is the average diameter. The sigmoid function, implemented in the tansig function in Matlab, was used as a transfer function between the neurons of all three layers [38].

The number of neurons in the hidden layer was found by increasing it till the ANN precision increased sufficiently, thus avoiding ANN over fitting behavior.

**Figure 2:**
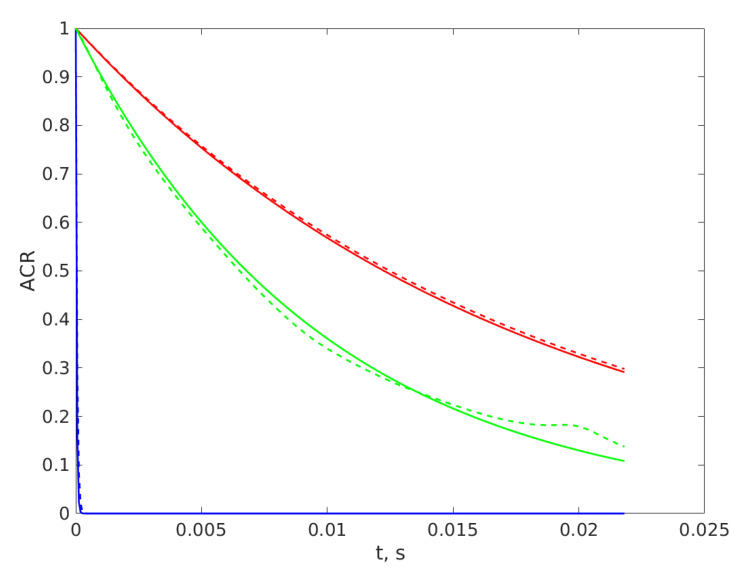
The autocorrelation (ACR) computed on time series generated without noise with Equation (7) for diameters of 10 nm, the continuous lower (blue) line; 15 nm, the lower dashed (blue) line; for 5100 nm, the continuous upper (red) line; and 5200 nm, the upper dashed (red) line. The ACRs for a diameter of 2858 nm computed with Equation (5) are presented with the continuous middle (green) line; and with noise added, Equations (10) and (11), with the dashed middle (green) line.

Figure 2 brings insight regarding the choice of the number of neurons in the input layer. As it was mentioned above, each value of the ACR, computed on each time series recorded with the same data acquisition frequency, is fed to a neuron in the hidden layer. 

Figure 2 reveals that the ACRs of the time series corresponding to very small diameters differ only at their first 4 lags, having values that are the same for the first significant four digits for a number bigger than 4 lags, therefore we cannot use averages for two, three, or more consecutive lags, as they will differ less. On the other hand, the ACRs for bigger diameters differ significantly from each other only at bigger lag number, being overlapped for the first two hundred lags, as can be seen in Figure 2, where the ACRs of time series for diameters significantly differ from each other, that is 0.1 µm, are plotted with the upper (red) lines. Taking a smaller number of lags for input, hence a smaller number of neurons in the input layer will both decrease the precision and limit the range of diameters that can be assessed using the ANN, because the differences between the data that is input to the ANN are significant only at bigger lag numbers.

The last layer has a single neuron, corresponding to the average diameter. 

Another thing that resulted from a trial and error procedure is that the best performance belonged to the ANN with training done on ACRs with noise added. The attempts at training the ANN with clean ACRs produced in a simple and tremendously less computation intensive manner, with Equation (5), led to very big errors when the inputs were ACRs of time series with noise added, as time series recorded in an experiment are, therefore this line was abandoned. This feature of the ANN can be explained by the distortion of the ACR produced by the added noise, as revealed by Figure 2, where the green lines present the ACRs computed for the same diameter, with and without added noise. 

The training algorithm was Levenberg–Marquardt [39]. Training stopped after 65 iterations, as the R-value reached 1, when rounded to 5 digits. The training lasted for 85 min on a laptop with an Intel i7-7300 processor, using 70% of the data sets for training the ANN, 15% for testing, and 15% for validation.

Once the ANN was trained, the relative errors of the ANN on the whole set of generated time-series were computed using Equation (12):

(12) $$err=\frac{d_{ANN}-d_{ref}}{d_{ref}}\cdot 100\left(\%\right)$$

**Figure 3:**
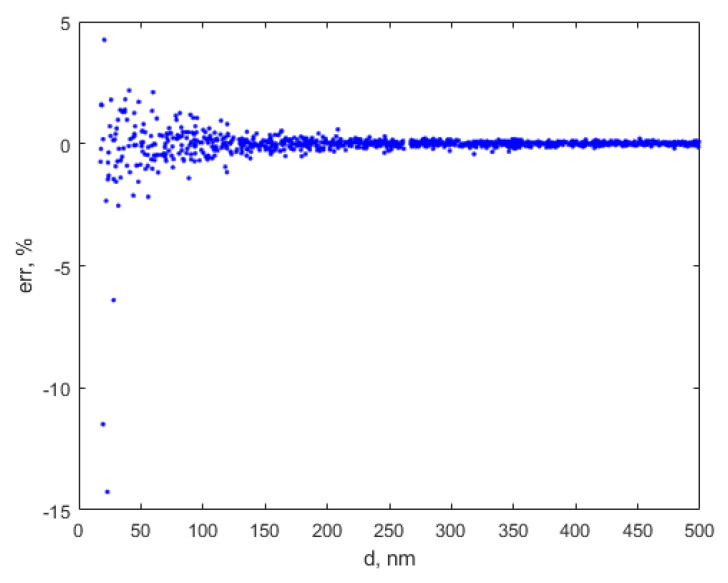
The variation of the relative errors for the diameter range [20–500] nm, computed with ANN.

As the training data set covered an extended diameter range, the relative errors computed with Equation (12) are very small for the diameter range 500–6000 nm, but are slightly bigger than the relative errors reported by [21] in the very small diameter range. Actually, the relative errors remain smaller that 0.1% for diameters bigger than 500 nm, therefore the whole range is not presented in Figure 3, but a zoom focusing on the 20–500 nm diameter range.

To conclude this brief error analysis paragraph, the ANN described in this work predicts the average diameter with a relative error up to 2.5% if the particles have a diameter in the range 25–70 nm, less than 1% if the particles have a diameter in the 70–150 nm range, less than 0.5% if the particles have a diameter in the 150–350 nm range, and less than 0.1% for particles having a diameter in the 350–6000 nm range. 

Moreover, we notice that the ANN assisted diameter assessment procedure is stable and predictable for an extended particle range, from 25 to 6000 nm. The results are not precise for suspended particles with a diameter smaller than 25 nm, as they can contain relative errors in the range from −14 to 4%, therefore it should not be used to assess the size of the particles in this diameter range. 

Furthermore, we should bear in mind that the average diameter mentioned in this work should be viewed as the hydrodynamic diameter, as it results from Equation (6), regardless of whether it is assessed by a least square minimization fit of Equation (5) or as a result of the ANN trained using simulated ACRs. Particles of nonspherical shape, like nanorods (not the case in yeast cells though) diffuse in a solvent and, when subjected to a DLS experiment, produce a time series and therefore a hydrodynamic diameter, considered an average diameter in the DLS sense. The diameter that is the output of the ANN procedure should be viewed as the diameter of spherical particles that diffuse in the same manner as the particles that produce a time series with the same ACR. Moreover, the diameter that is assessed by the ANN assisted DLS described in this work is slightly different from the diameter that can be assessed by other physical procedures, like optical microscopy, transmission electron microscopy static light scattering, atomic force microscopy with its different mode of operation, and others; a detailed explanation of the differences can be found in [11], on a crystalline substance, as Fe_3_O_4_ nanoparticles, where X-ray diffraction can be an option. Optical microscopy, using visible light with a wavelength in the range 0.380–0.740 μm, cannot be expected to produce precise results on particles with the size of a few μm, because the light wave diffraction will manifest. On the other hand the DLS techniques has been validated decades ago [4], therefore the DLS was used as reference.

**Figure 4:**
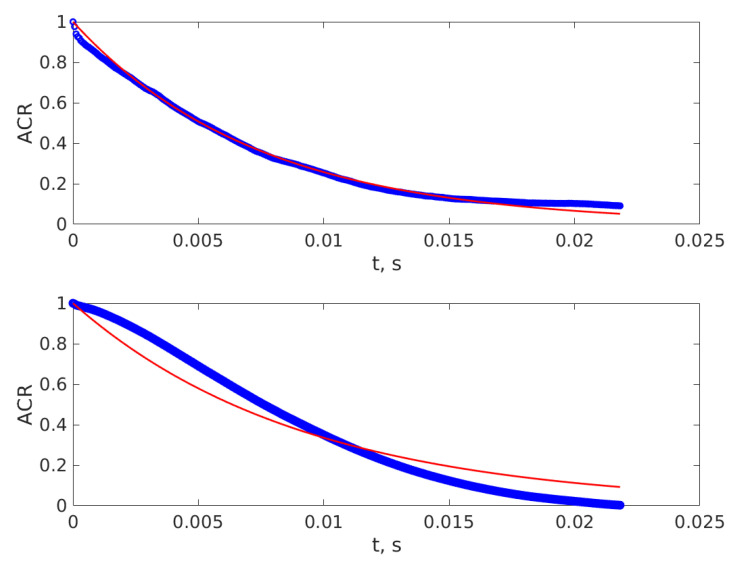
The ACR of two of the time series. The upper subplot illustrates an ACR that is well described by Equation (5) and the lower subplot the ACR that is the most departed from the ideal exponential decay described by Equation (5).

If the suspended particles have a wider size distribution, the ACR is actually a sum of ACRs, well described by Equation (2), but that cannot be described by Equation (5). For the case of yeast cells examined during fermentation the approximation of Equation (5) holds to a certain degree, as illustrated by Figure 4. The upper subplot illustrates one of the ACRs that is very well described by Equation (5), while the lower subplot illustrates one of the ACRs that is described worse by the simple exponential decay of Equation (5).

The distortion from the simple exponential line at bigger lag numbers is caused by the noise in the experimental time series, which is confirmed by ACRs of the simulated time series, illustrated in Figure 2. The middle lines of Figure 2 illustrate the ACR for the ideal mono-dispersed spheres and the ACR of the same time series after noise addition, as described above, in this Section. We notice that the noise increases the ACR at bigger lag numbers, which is the same feature displayed by the ACR of the experimentally recorded time series presented in the upper subplot of Figure 4. Moreover the upper subplot of Fig. 4 reveals a decrease of the ACR that is faster than the simple exponential decrease, which can be caused by the smaller particles in the yeast cell group. The glucose and fructose molecules, having diameters smaller than the lower diameter range that can be detected by the procedure that is described in this work due to the lower acquisition frequency used, might be a cause for the distortion in the small lag number of the ACR as well.

**Figure 5:**
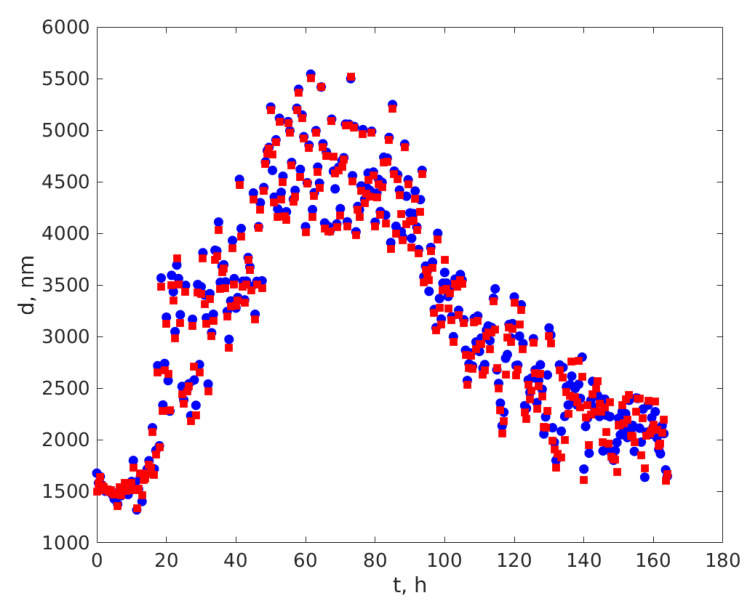
The average yeast cell diameters during fermentation. The circles depict diameters assessed using the reference procedure and the squares for the diameters assessed using the ANN assisted procedure.

The fact that some ACRs are well described and others are not so well described by a simple exponential decay might be the explanation for the fluctuations of the predicted diameters by both the reference and the ANN procedure, described by Figure 5. The decrease in precision is a consequence of the simplicity of both the experiment and of using the description for mono-dispersed particles in training the ANN, resulting in a simple function with the ACR as input and the average hydrodynamic diameter as output. Moreover, as the yeast cell concentration increases, conditions for restricted diffusion might occur and the predicted diameter might be slightly bigger than the real diameter, and this might be another cause of possible errors and fluctuations.

Another question that can rise from ACR examination is related to the origin of the suspended cells motion. If particles the size of microns have a density different from the density of the solvent they undergo sedimentation, and in a matter of a few hours the particles can be either on the bottom or at the surface of the cuvette. This was not the case after 164 h though; therefore, the scattered light fluctuations were produced by the yeast cells’ Brownian motion, causing diffusion.

### 2.4. Experimental Procedure and Time-Series Processing

Matlab code was written and used to record the DLS time-series. The length of the time-series and the delay between consecutive recordings are adjustable parameters.

The experiment lasted for 164 h. A time series lasting for 8 s was recorded every 30 min. Yeast fermentation was initiated in the circular cuvette containing the sucrose solution, as described in Section 2.1. After the experiment ended and data acquisition was completed, the time series were stored in an array, the ACRs for them were computed and the first 350 lags were added as a column to the ACRs array. The ACRs array was used later on as input to the Matlab function having the weights and biases, as generated by the training procedure, contained by the code (hardcoded) and the average diameters were computed as the output of the function corresponding to ANN, as described in Section 2.3.

## 3. Results

The average diameters for each time-series were computed using the ANN designed and trained as described in Section 2.3 and are plotted versus t, the time elapsed from the beginning of the first recording in Figure 5. The blue circles represent the diameters assessed using the reference procedure and the squares for the diameters assessed using the ANN assisted procedure.

**Figure 6:**
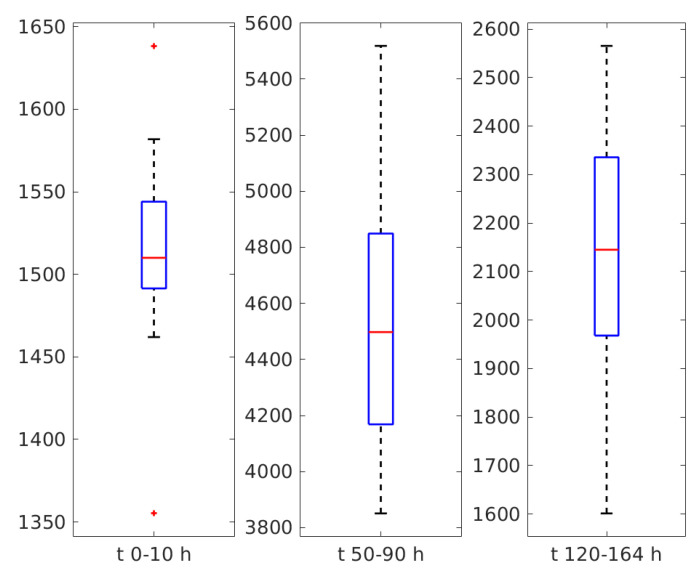
The boxplot of the diameters, in nm, on the three plateaus: 0–10 h, 50–90 h and 120–164 h.

Another way of looking at the diameters of the particles in suspension is to render them as a boxplot. The diameters of the three time intervals that present a plateau, with fluctuations though, were grouped and presented as a boxplot for each group in Figure 6.

The fluctuations might be fitting artifacts, both of the reference and of the ANN assisted ACR’s processing procedures, as the particles in suspension are not mono-dispersed. Examining Figure 5 and Figure 6 we notice that during the time interval 0–10 h the average diameter was around 1510 nm, during the 50–90 h plateau around 4450 nm, and during the 120–164 h plateau around 2150 nm.

Additionally, Figure 5 depicts that the diameters computed using the two procedures are very close to each other, which is consistent with the relative error computed on the simulated time series, as presented in Figure 3. 

We also notice that at the beginning of the experiment the size of the particles remained constant for 10 h, with fluctuations. The increase of the diameter occurred after about 10 h. 

Yeasts may have asexual and sexual reproductive cycles, a feature that is typical for fungi. Reference [40], as many others, states that the most common mode of population growth in yeast is asexual reproduction by budding. During budding, a small bud is formed on the parent cell and during this process the nucleus of the cell splits and migrates into the daughter cell [40]. The bud continues to grow and separates from the parent cell, forming a new cell [41] which is smaller than the mother cell and continues to grow after separation. This stage of yeast cell population evolution is consistent with the increasing of the average diameter of some of the SCs during the time interval from 10 to around 50 h since the beginning of the experiment. During that time interval an increasing number of yeast cells were consuming glucose and were multiplying. Their assessed diameter was expected to increase, as the diffusion coefficient decreased, because the volume and hence the equivalent hydrodynamic diameter of the mother and daughter cell prior of separation was bigger than the hydrodynamic diameter of a single cell. The average diameter remained on a plateau for up to 90 h from the beginning of the experiment, as the yeast cells continued to consume glucose and to multiply by budding. We also notice from Figure 5 and Figure 6 that the average diameter exhibits fluctuations around 4450 nm. This value of the assessed diameter is consistent with the electron microscope assessment of the *S. cerevisiae* size reported in [42]. Reference [43] states that the yeast cells are ellipsoidal having approximately 4.76 μm on the long axis and 4.19 μm on the short axis for haploids and 6.01 by 5.06 μm for diploids. We should also note that electron microscopy and DLS are different physical procedures and, therefore, the diameter assessed using these different procedures are expected to be different from each other [11,44].

The diploid yeast cell under nutritional starvation gives rise to four haploid meiotic progeny, each of them being encapsulated in a spore. All four products from a single meiosis are wrapped together in a sac, called an ascus [42]. This might be the situation in the small cuvette volume after about 90 h since the beginning of the experiment with the glucose having been consumed by the yeast cells. This is consistent with the decrease of the average diameter as assessed by both the reference procedure and the ANN assisted procedure. The average diameter continued to decrease as the number of haploid cells increased and the number of diploid cells decreased.

Figure 5 also indicates that after about 140 h the diameter remains around 1950 nm. This average diameter is considerably smaller than the size of the haploid cells. Reference [42] indicated that the ascus wall can be digested by degradative enzymes to separate the individual spores from each other. A possible explanation of the decrease of the average diameter to such small values, smaller than the reported size of the haploid cells [42], might be that the ascus wall was disrupted and that the individual spores became free and suspended in the cuvette. 

Coming back to the first plateau and the last plateau, corresponding to the time interval 0–10 h, we notice the median values of the assessed diameter are 1510 nm and 2150 nm, respectively, considerably smaller that the diameter of both diploid and haploid yeast cells. If we make a rough estimation of the volume of the haploid cell, considering it to be a sphere with an average diameter of 4500 nm, and we divide it by four and assess the diameter of such a smaller sphere we get a value of roughly 2800 nm. This value is slightly bigger than what was measured using both the ANN assisted DLS and the reference procedure on both plateaus. A possible explanation is that when haploid cell split into spores, each spore does not receive a quarter of the haploid cell volume, but receives less. The first plateau corresponding to the time interval 0–10 h from the beginning of the experiment presented a median value of 1510 nm, smaller than the median diameter of the last plateau. A possible explanation might be that in its solid form, in the sample that was used, the yeast was in the form of spores and during the first 10 h the spores hydrated and arrived at a condition to recombine forming living yeast cells.

## 4. Discussion and Conclusions

This paper presents an ANN assisted procedure for DLS time series processing, with details on the ANN design and training procedure. The procedure has the average diameter of the suspended particles as output. This average diameter should be viewed as a DLS average diameter, which is the diameter of mono-dispersed particles that produce a DLS time series with the same ACR. The upper range for the particle size that can be precisely measured with the ANN assisted procedure has been increased from several hundred nanometers in the version presented in [20], and 1200 nm in the version presented in [21], to 6000 nm in this version. Moreover, an error analysis of this ANN assisted procedure, with respect to the reference method described in Section 2.2, was carried out over an extended particle size range, which is from 25 to 6000 nm. The relative error, as defined in Equation (12), is up to 2.5% if the particles have a diameter in the 25–70 nm range, less than 1% if the particles have a diameter in the 70–150 nm range, less than 0.5% if the particles have a diameter in the 150–350 nm range, and less than 0.1% for particles having a diameter in the 350–6000 nm range.

The ANN assisted DLS time series processing, working on an extended diameter range, remains considerably faster than the reference DLS time series processing procedure described in Section 2.2, thousands of times faster actually, which, in its turn is faster than the reference procedure described in [20], because it has one free parameter, instead of two. Stating a precise number for how many times the procedure is faster would be imprecise, because the time for completing the reference procedure strongly depends on the starting parameter (educated or less educated guess) and of the random parameters used by the least squares procedure itself. For example, the whole set of 329 time series recorded of the yeast suspension mentioned in this work were processed using the reference DLS procedure in 1.3166 s. The ANN assisted DLS procedure lasted for 2.0837 × 10^–4^ s. Both times refer to processing the ACRs already computed and loaded in computer RAM as variables. The outputs of both procedures were retained as variables as well, thus not involving hard disk operations. The ANN assisted DLS procedure was therefore 6319 times faster than the reference DLS, for the above mentioned set of data. At this point it is worth mentioning that being faster is not a big advantage for the type of experiment mentioned in this work, which lasted for 164 h, but might be of interest for processes that complete in a matter of seconds, like nanoparticle aggregation, as reported in works like [1,2,43,44].

Moreover, the procedure can be made thousands of times faster if no output is written on the computer hard drive, but retained as variable. The time for recording the time series remains the same though, and the above mentioned statement is true for the time required for processing the DLS time series once recorded.

The ANN assisted DLS time series processing presented here is not intended to replace the CONTIN [45] or the maximum entropy methods, it is just another approach for time series processing. CONTIN relies on the inverse Laplace transform, being basically an improvement of the inverse Laplace transform method. Calculating the inverse Laplace transform is problematic on experimental data and can lead to ambiguous results, this being an ill-posed mathematical problem. The CONTIN method tries to counter this effect by introducing regularization, driven by a parameter which has a very big influence on the solution found. The maximum entropy method assigns entropy to the solutions and searches for the solution with maximum entropy, which is computation intensive, as well. The ANN based DLS, with its limitations of providing the average DLS diameter only, as described in this work, was designed to avoid these complications. Moreover, this procedure is a step forward in designing the ANN assisted DLS, a pure computational procedure, which will have the size distribution as output, without any assumption regarding the theoretical function to describe it. The method, as presented, is less computation intensive than fitting a function to a set of data because the ANN procedure actually consists of matrix multiplication and addition. It is easy to implement on a low power computation platform like a cell phone or development platform, as it does not require particular libraries with the functions required for least squares methods.

The method, simple to use once the ANN has been trained, was used to monitor the variation of the average *S. cerevisiae* yeast cell size during population evolution since dissolving from solid form in sucrose solution till nutrition stress occurrence. The stages of the cell population evolution, as hydration, multiplication by budding, haploid formation, and spore releasing were made evident by the variation of the average diameter assessed using the ANN assisted DLS procedure. With these aspects in mind, the experiment presented in this work can be considered a proof of concept for the proposed method, easy to use and low on resources, which qualifies the simple setup and the procedure to be a possible sensor for different processes in food industry where the size of the suspended particles is related to different stages of the process. Moreover, this simple procedure can be used to identify the presence of bigger particles, like bacteria, in a suspension where the expected particles are in much smaller diameter range, which might be useful in establishing a fast procedure for safety or quality assurance.
